# Ultrasonographic and Scintigraphic Findings of Thyroid Hemiagenesis in a Child: Report of a Rare Male Case

**DOI:** 10.1155/2015/917504

**Published:** 2015-02-15

**Authors:** Ümit Yaşar Ayaz, Sevin Ayaz, Mehmet Ercüment Döğen, Arman Api

**Affiliations:** ^1^Mersin Kadin Dogum ve Çocuk Hastaliklari Hastanesi, Radyoloji Bölümü, Halkkent, Toroslar, 33240 Mersin, Turkey; ^2^Mersin Devlet Hastanesi, Nükleer Tip Bölümü, 33050 Mersin, Turkey; ^3^Mersin Kadin Dogum ve Çocuk Hastaliklari Hastanesi, Çocuk Cerrahisi Bölümü, Halkkent, Toroslar, 33240 Mersin, Turkey

## Abstract

Thyroid hemiagenesis is a rare congenital anomaly in which one lobe of thyroid gland fails to develop. It is much rarer in males. There is a higher incidence of associated thyroid disorders in patients with thyroid hemiagenesis; therefore early and prompt diagnosis is important for children. We present the ultrasonographic and scintigraphic findings of thyroid hemiagenesis in an eight-year-old-boy. On ultrasonography (US), left lobe of the thyroid gland could not be demonstrated and the right lobe showed minimal hyperplasia. Its echogenicity was normal and no nodule was seen. On thyroid scintigraphy, left lobe of thyroid gland or any ectopic thyroid tissue could not be demonstrated, while the right lobe showed minimal hyperplasia. Without performing any invasive procedure, we enrolled the child in a follow-up program with the guidance of US and scintigraphy, which were effective both in making the final diagnosis of thyroid hemiagenesis and in evaluating the current status of the present thyroid tissue. In conclusion, if only one thyroid lobe is detected in a pediatric case initially with US or scintigraphy, the diagnosis of thyroid hemiagenesis should be suggested and, before any unnecessary or invasive attempt, the other complementary method (scintigraphy/US) should be performed.

## 1. Introduction

Thyroid hemiagenesis is a rare congenital anomaly in which one lobe of thyroid gland fails to develop. Isthmus may be present or absent [1]. The true prevalence of thyroid hemiagenesis is not exactly known. Its prevalence was reported as 0.05%–0.2% in asymptomatic and/or normal pediatric population [2–4]. Gursoy et al. [5] reported the prevalence of thyroid hemiagenesis as 0.25% in patients with thyroid disorders and as 0.025% in normal population. In an endemic area where goiter and thyroid nodules were frequent, Aydin et al. [6] reported two cases of thyroid hemiagenesis out of 2233 adults, with a percentage of about 0.09%. Though Maiorana et al. [3] reported slightly higher incidence of thyroid hemiagenesis among male children, female predominance was reported in other studies [1, 2, 4, 7, 8]. Thyroid hemiagenesis is much rarer in males and our purpose is to present the ultrasonographic and scintigraphic findings of thyroid hemiagenesis in a male pediatric case.

## 2. Case Presentation

**Figure 1:**
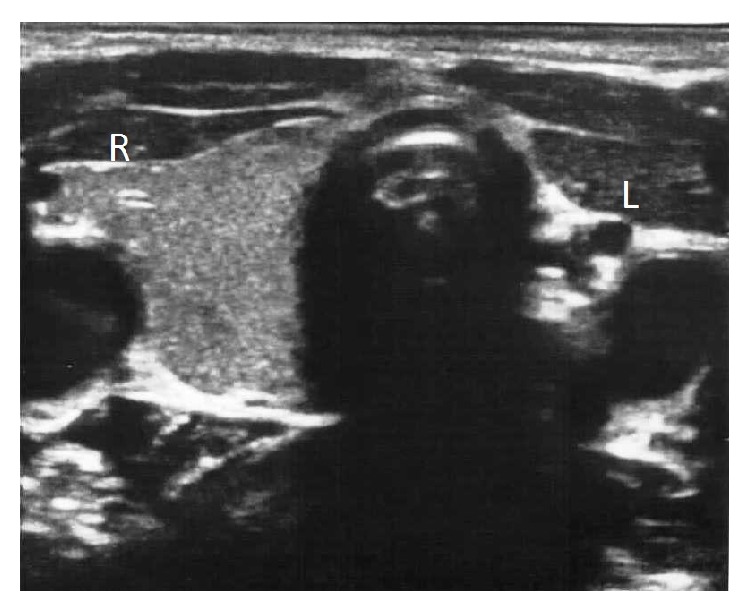
On transverse sonogram of the thyroid, right lobe (R) displays normal, homogeneous echotexture without any nodule. Left lobe cannot be demonstrated. Strap muscles and vascular structures are displaced to fill the location of the left lobe (L).

**Figure 2:**
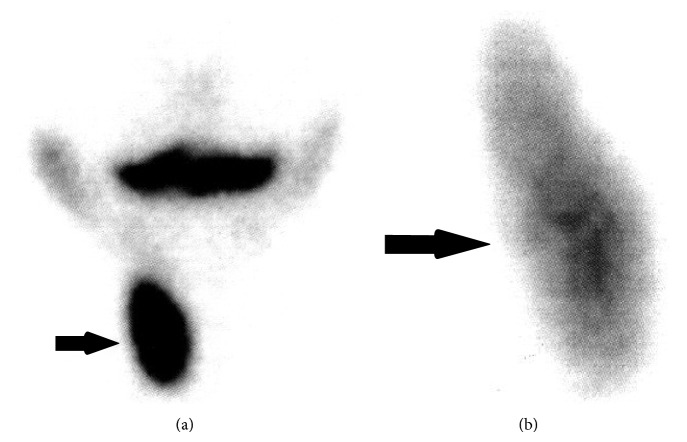
Scintigraphic images of the neck obtained by parallel-hole collimator (a) and pinhole collimator (b). On anteroposterior views, right lobe of the thyroid gland (black arrows) shows minimal hyperplasia with homogeneous uptake. No nodule is demonstrated. At the location of the left lobe of thyroid gland and any other parts of the neck, no accumulation of radioactivity is obtained.

An eight-year-old boy with a history of a self-limited head trauma last year seemed restless and rather unable to stand still during inspection. History of the patient revealed no medical treatment related with thyroid disorders and no history of neck irradiation or thyroid surgery. There was no family history related with any of the thyroid diseases. Physical examination was normal and no congenital anomaly was demonstrated. The thyroid gland was unpalpable. The child was 120 cm in height and weighed 21.5 kg. He was euthyroid, FT4: 1.00 ng/dL (range, 0.70–1.48 ng/dL) and TSH: 3.2 *μ*IU/mL (range, 0.35–4.94). Thyroid ultrasonography (US) was performed in supine position with a Mindray DC-7 US device (Shenzhen Mindray Bio-Medical Electronics Co., Ltd., Shenzhen, China), using 8 MHz linear-array transducer. The length, width, and depth of the right lobe were measured, and the volume was calculated by the mean of the elliptical shape volume formula (*π*/6 × length × width × depth) [6, 9]. The dimensions of the right lobe were 37.1 × 14 × 12.5 mm and its volume was 3.4 mL revealing minimal hyperplasia, as the age, gender, weight, and height of the child were of concern [4]. The echotexture of the right lobe was normal and homogeneous. Its margins were regular. Anterior to trachea, a thin, band-like image isoechoic to the right lobe, measuring 1.7 mm in thickness and resembling a thin isthmus, was noticed; however a left lobe could not be demonstrated. Strap muscles, left common carotid artery, jugular vein, and other regional vascular structures were displaced to fill the location of the left lobe (Figure 1). On US examination of other parts of the neck, pyramidal lobe, any midline cyst such as thyroglossal cyst or any mass consistent with an ectopic thyroid tissue such as lingual thyroid could not be demonstrated. For ruling out any ectopic thyroid tissue, thyroid scintigraphy was performed by intravenously administering 2.5 mCi Tc-99m pertechnetate. Images of the head and neck were taken 20 minutes later with dual-head gamma camera (Siemens e-cam, Erlangen, Germany), using parallel-hole and pinhole collimators. Additionally, planar scintigraphic imaging of thorax, abdomen, and pelvis was performed. Scintigraphically, right lobe of the thyroid gland showed minimal hyperplasia, though uptake was homogeneous. Tc-99m uptake of the right lobe was 3.6% (normal range, 0.3–3%). No nodule was demonstrated at the right lobe. At the location of the left lobe of thyroid gland and any other parts of the neck, no accumulation of radioactivity could be obtained (Figures 2(a) and 2(b)). Thoracal, abdominal, and pelvic scintigraphic imaging revealed no ectopic thyroid gland activity.

## 3. Discussion

Embryologically, the thyroid gland develops at the junction between tuberculum impar and hypobranchial eminence which forms foramen caecum [10]. With further development, the gland descends in front of the hyoid bone and the laryngeal cartilages, reaching its final position in front of the trachea [10]. The etiology of thyroid hemiagenesis is still unclear; however it was reported that the absence of compensatory growth of the other lobe was suggestive of a lobulation defect rather than a local problem interfering with the descent [4].

The term thyroid dysgenesis expresses a wide range of anomalies including athyreosis and thyroid hypoplasia (global hypoplasia and thyroid hemiagenesis) [11]. Thyroid hemiagenesis was included in the group of mild thyroid developmental anomalies such as pyramidal lobe and thyroglossal duct cysts [11]. In thyroid hemiagenesis, involvement of the left lobe is abundantly more frequent [1–4, 7, 8].

Without referring to recent large scale studies [5–8], thyroid hemiagenesis could have been considered as a rather benign congenital anomaly requiring no treatment, since a single thyroid lobe is sufficient to maintain clinical euthyroidism [7]. Gursoy et al. [5] reported that the prevalence of thyroid hemiagenesis in patients with thyroid disorders is tenfold greater than in normal population. A large cohort case-control study conducted by Ruchala et al. [7] revealed a higher incidence of associated functional, morphological, and autoimmune thyroid disorders in patients with thyroid hemiagenesis, the most frequent disorders being thyroid nodules and autoimmune thyroid diseases, when compared to subjects with bilobate thyroid glands. Therefore early and prompt diagnosis is important to enroll the child in a follow-up program. Ultrasonography (US) and radionuclide thyroid scanning are the imaging modalities of choice in the evaluation of the thyroid gland in children [11]. Fine-needle aspiration biopsies, laboratory tests, and also scintigraphies are useful to diagnose other diseases within the remaining lobe [1, 7].

In the present case, US gave us all the morphological data which we needed to make the prediagnosis of thyroid hemiagenesis and to evaluate the features of present thyroid lobe and isthmus. Other parts of the neck were also evaluated for any mass consistent with an ectopic thyroid tissue. However, to make an exact diagnosis and to know whether there was any ectopic thyroid tissue in other parts of the body, we had to perform thyroid scintigraphy. We also wanted to evaluate the uptake of the present thyroid lobe. In our case, sonographic examination using a high frequency linear-array probe and thyroid scintigraphy with Tc-99m were both essential and complementary in the diagnosis of thyroid hemiagenesis.

In conclusion, if only one thyroid lobe is detected in a pediatric case initially with US or scintigraphy, the diagnosis of thyroid hemiagenesis should be suggested and, before any unnecessary or invasive attempt, the other complementary method (scintigraphy/US) should be performed for final diagnosis.
